# Supplementary material for: Yiai Fuzheng decoction inhibits triple-negative breast cancer by remodeling the immune microenvironment
Source: Front Immunol. 2025 Sep 30;16:1615631. doi: 10.3389/fimmu.2025.1615631 (PMC12518410; doi:10.3389/fimmu.2025.1615631)
Supplement: Supplementary file 2 [file DataSheet1.zip › Supplementary File 2/Treatment/C_vs_D/07_Pathway_Analysis/Pathway_Analysis/Sig_Pathways_ID/Amino sugar and nucleotide sugar metabolism.pdf]

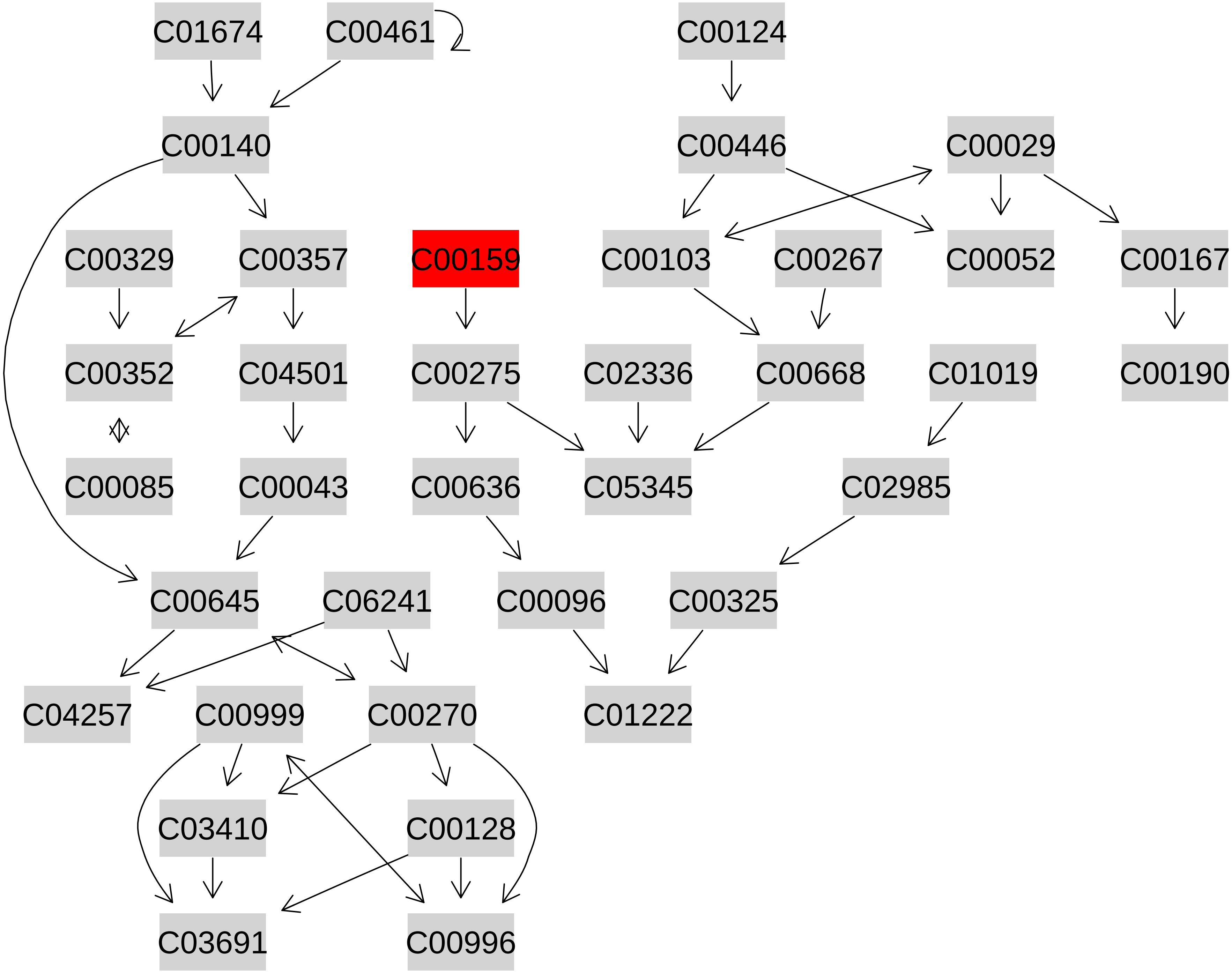
C01674
C00461
C00124
C00140
C00446
C00029
C00329
C00357
C00159
C00103
C00267
C00052
C00167
C00352
C04501
C00275
C02336
C00668
C01019
C00190
C00085
C00043
C00636
C05345
C02985
C00645
C06241
C00096
C00325
C04257
C00999
C00270
C01222
C03410
C00128
C03691
C00996
